# Does transdermal fentanyl work in patients with low BMI? Patient‐reported outcomes of pain and percent pain relief in cancer patients on transdermal fentanyl

**DOI:** 10.1002/cam4.2479

**Published:** 2019-09-30

**Authors:** Natalie Moryl, Ali Bokhari, Yvona Griffo, Rachel Hadler, Lauren Koranteng, Alexandra Filkins, Tianyu Zheng, Susan D. Horn, Charles E. Inturrisi

**Affiliations:** ^1^ The Palliative Medicine Memorial Sloan Kettering Cancer Center New York NY USA; ^2^ Health System Innovation and Research Division University of Utah School of Medicine Salt Lake City UT USA; ^3^ Department of Pharmacology Weill Cornell Medical College New York NY USA

**Keywords:** cancer management, clinical observations, medical oncology, nutrition

## Abstract

**Background:**

Low body mass index (BMI) is suspected of being associated with low transdermal fentanyl (TDF) blood levels and worse pain relief. Clinical pain data to support this claim are lacking.

**Methods:**

Using a Chronic Pain Registry, we identified 901 cancer patients who received TDF at outpatient pain service clinics of our cancer center from 7/1/2011 to 12/1/2016. Of these, 240 patients had a BMI measure, pain intensity, and pain relief scores documented within 30 days of a TDF order. We examined associations between BMI, TDF dose, Worst and Least pain scores, and pain relief scores using standard statistical tests.

**Results:**

In cancer patients receiving TDF, low BMI (<18.5) was significantly associated with greater pain relief irrespective of TDF dose and borderline significantly associated with greater percent pain relief after controlling for age, cancer diagnoses, and pain etiology (*P* = .073), suggesting that low BMI may independently predict better pain relief in cancer patients. As there were no significant associations between BMI and TDF dose, we find no basis for BMI‐dependent dose modification or avoiding TDF in cachectic and low BMI patients.

**Conclusions:**

When predicting percent pain relief, we conclude that there is no basis for avoiding TDF or modifying its dose in cancer patients with low BMI and cachexia.

## Graphical abstract

Low body mass index (BMI) is suspected of being associated with low transdermal fentanyl (TDF) blood levels and worse pain relief. Of the 240 patients who reported patient‐reported outcomes, whereas on TDF, patients with lowest BMI trended toward better pain relief on the lowest TDF doses. When predicting percent pain relief, we found no basis for avoiding TDF or modifying its dose in cancer patients with low BMI and cachexia.

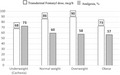

## 1 BACKGROUND

Transdermal fentanyl (TDF) is one of the most common opioids prescribed to patients with cancer.^1^, ^2^, ^3^ TDF offers multiple advantages compared to other opioids. TDF has lower incidence and severity of adverse effects such as constipation, nausea and vomiting, and daytime drowsiness. Patients report both greater satisfaction and improved quality of life. Administration every 72 hours favors improved convenience and compliance. In addition, patients receiving TDF had less use of rescue medications.^4^

Transdermal fentanyl may be useful especially in patients with chemotherapy and radiation‐induced mucositis, gastrointestinal obstruction, patients with other gastrointestinal problems that may interfere with absorption, and patients who are unable to swallow. It is also advantageous for patients who forget to take their prescription medications, have dementia or cognitive impairment from brain metastases, as well as for patients for whom there is concern about compliance. Many of these factors are seen in patients with low body weight during treatment (eg, cachexia during radiation for head and neck cancer) and in advanced cancer patients with high prevalence of weight loss and cachexia.

Previously, delivery rate‐adjusted serum fentanyl concentration has been shown to vary widely.^5^, ^6^ Many studies explored clinical and genetic factors responsible for such variability. Fentanyl is mainly metabolized in the liver by CYP3A4 into inactive metabolites and fentanyl clearance varies greatly in liver disease.^6^, ^7^ Elimination of fentanyl and metabolites is not influenced by moderate to severe renal impairment.^6^, ^8^ Age has been proposed to be a factor in different phases of pharmacokinetics,^9^, ^10^ however, the overall influence of age is thought to be insignificant in clinical practice.^11^ Studies do not support significant influence of gender on TDF absorption.^6^, ^8^, ^9^, ^12^, ^13^ Hyperhidrosis, hypertrichosis, and localization of patches on the skin were found to not affect bioavailability of TDF.^10^ Effects of low albumin on serum fentanyl concentrations at 9‐24 hours after application of the patch^13^ were not found to be relevant in a large cross‐sectional clinical study.^6^

Body mass index (BMI) has been suspected of being associated with lower fentanyl blood levels in cachectic patients.^14^ In a small 3‐day prospective study of 10 cachectic and 10 normal weight cancer pain patients, mean TDF dose in patients with normal body weight was more than double (86 ± 29 mcg/h) of what it was in cachectic patients (42 ± 10 mcg/h). Although the number of evaluable subjects was 18 and pain report was similar among the cachectic and normal weight subjects, this report has been cited as a reason to avoid TDF in patients with low BMI. This concern has influenced clinical practice and guidelines.^15^ Later studies of residual fentanyl remaining in fully used TDF patches showed no associations with BMI.^8^, ^11^ In a recent comprehensive study of 620 cancer pain patients, less than 50% of variability in serum fentanyl concentrations was accounted for by combined CYP3A4/5 genotypes and clinical variables including dose, sex, co‐medications, kidney disease, BMI, and serum albumin.^6^ In contrast to the previous prospective small study showing an association of higher BMI with higher serum fentanyl concentration,^14^ the authors of a large 620 patient study found that higher BMI was (weakly) associated with lower serum fentanyl concentrations.^6^ Regretfully, this large comprehensive study didn't report pain or pain relief outcomes.

In a recent study of 129 cancer pain patients undergoing opioid rotation from strong opioids to TDF, the equianalgesic ratio was not significantly impacted by BMI or serum albumin.^1^ The authors however excluded 41% of patients whose pain escalated. Data on how BMI affects clinical pain control (pain levels and percent pain relief) in patients on TDF are lacking. The purpose of this study was to determine the associations between two different classifications of BMI, the TDF dose, Worst and Least pain scores, and pain relief and ultimately whether there is a basis to avoid TDF in patients with low BMI or for a BMI‐dependent dose modification of TDF dosing in cancer patients.

## 2 MATERIALS AND METHODS

The Institutional Review Board at Memorial Sloan Kettering Cancer Center approved the Patient‐Reported Outcomes (PRO) Pain Registry project that included patients seen in outpatient pain clinics between 7/1/2011 and 12/1/2016.

### 2.1 Patient assessments

We collected information regarding patient demographics, TDF dose, and BMI from the electronic medical record. Among the PRO, Worst and Least pain intensity scores, and percent pain relief were collected as part of the Brief Pain Inventory.^1^, ^16^ Cancer diagnoses were classified according to the American Cancer Society cancer classification, 2018, American Cancer Society.

### 2.2 Eligible patients

Patients over 18 years old who completed PRO including Worst and Least pain and percentage pain relief within 30 days after receiving TDF prescription and with BMI recorded within 30 days were included in the analysis. As each patient received TDF for at least 30 days prior to completing PRO we assume that the patients had come to steady state.^17^

### 2.3 Statistical methods

Our primary objective was to evaluate the correlations between BMI, TDF dose, Worst and Least pain, and pain relief.

Two different classifications of BMI were used:

1. Five BMI categories (<20, 20‐21.9, 22‐24.9, 35‐27.9, ≥28), described in a recent study of BMI as a prognostic indicator in cancer patients.^18^
2. Four BMI categories (underweight or BMI of <18.5, Normal weight or BMI of 18.5 to <25, Overweight or BMI of 25.0 to <30, and obese or BMI ≥30) used by Center for Disease Control (CDC).^19^

Data were summarized first using standard descriptive statistics. Then, associations between categorical variables were examined by chi‐square tests or Fisher exact tests. Kruskal‐Wallis nonparametric one‐way analysis of variance test was used to examine differences in continuous variables between four or five BMI groups. Correlations were assessed using the Spearman correlation coefficient. Linear regression models were applied to estimate the linear associations between predictor variables of BMI categories and TDF dose only and dependent variables. Subsequently, regression models were re‐analyzed adding the covariates of Age, Cancer Diagnosis, and Pain Etiology (cancer pain vs. cancer treatment‐related pain in a patient with cancer in remission) to the regression models with BMI categories and TDF dose. All *P* values < .05 were considered statistically significant. All computations were carried out in SAS software (version 9.4; SAS Institute Inc, Cary, NC).

## 3 RESULTS

Of 2320 cancer pain patients that took part in the prospective PRO project in pain clinics of a tertiary cancer center between 7/1/2011 and 12/1/2016, 901 patients received TDF. Of these, 240 patients satisfied inclusion criteria of having three measures of interest: a BMI measure, a TDF dose recorded within 30 days of a Worst and a Least pain intensity score, and a percent pain relief score.

Of 240 patients included in analysis, 124 were females, 193 self‐reported their race as white, 27 as black, 7 as Other, Asian or Native Indian, and 13 refused to answer. Median age was 60 with interquartile range of 52‐70 and full range from 22 to 98. Cancer diagnoses classified according to the American Cancer Society cancer classification, 2018, American Cancer Society, Inc, Surveillance Research are listed in Table 1.

**Table 1:** Cancer diagnoses based on the American Cancer Society cancer classification, 2018, American Cancer Society, Inc, Surveillance Research

Oral cavity & pharynx	19
Digestive system	43
Esophagus	4
Stomach	1
Colon	11
Rectum	7
Anus	3
Liver and gallbladder	3
Pancreas	14
Respiratory system	
Larynx	1
Lung and other	32
Bones and joints	2
Soft tissue (including heart)	32
Breast	13
Genital system	26
Uterine cervix	3
Uterine corpus	7
Ovary	9
Prostate	13
Testis	4
Urinary system	29
Urinary bladder	13
Kidney and renal pelvis	16
Brain and nervous system	2
Endocrine system (including thyroid)	5
Lymphoma, leukemia, MDS	14
Myeloma	14
Other	5
No cancer diagnosis	5
Total	252

While generally consistent with the American Cancer Society and previously published result of all patients in our Pain Registry,^16^ not surprisingly, patient cohort treated with TDF had a higher rate of patients with oral cavity, pharyngeal, and laryngeal cancers.

**Figure 1:**
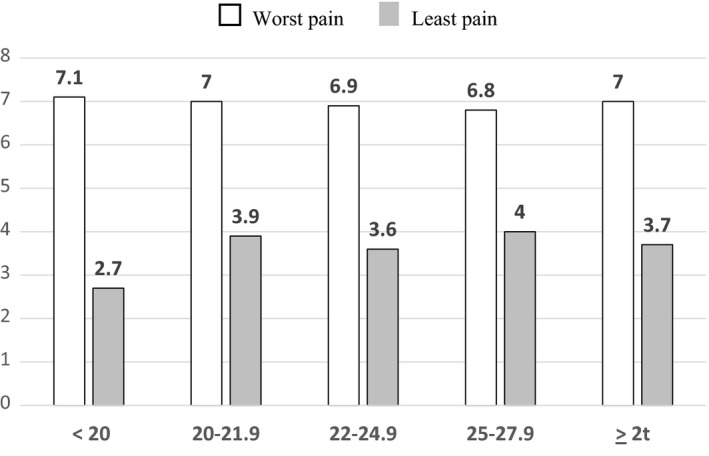
The Worst and Least Pain scores as a function of the body mass index (BMI), kg/m^2^ classification.^18^ □ indicates the Worst Pain on 0‐10, 11‐point Brief Pain Inventory (BPI) pain scale and 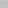 indicates the Least pain in the previous 24 h

**Figure 2:**
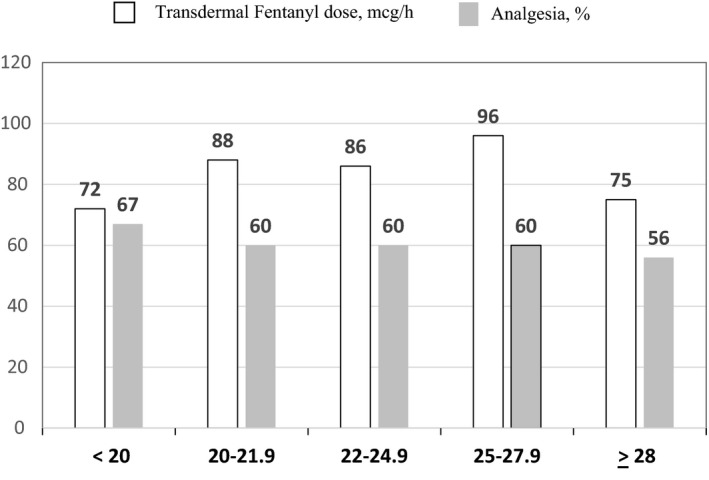
Transdermal Fentanyl dose, mcg/ and % Analgesia as a function of the body mass index (BMI), kg/m^2^ classification.^18^ □ indicates Transdermal Fentanyl dose, mcg/h and 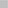 indicates percent Analgesia, % in the previous 24 h

**Figure 3:**
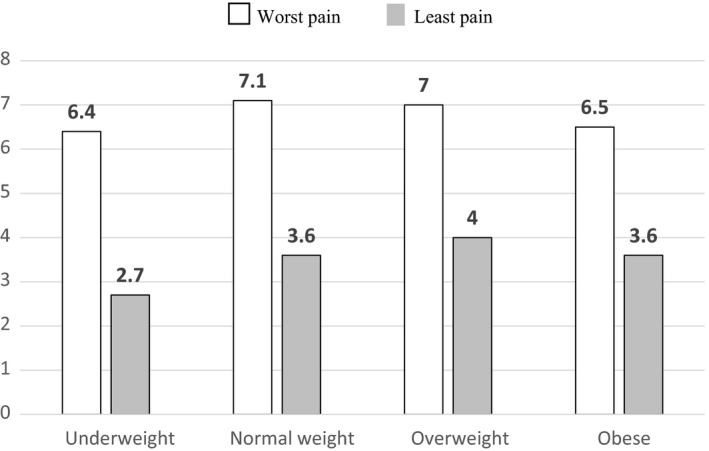
The Worst and Least Pain scores as a function of the body mass index (BMI), kg/m^2^ classification used by Center for Disease Control (CDC).^18^ Four BMI categories include underweight or BMI of <18.5, Normal weight or BMI of 18.5 to <25, Overweight or BMI of 25.0 to <30, and obese or BMI ≥30.^19^ □ indicates the Worst Pain on 0‐10, 11‐point Brief Pain Inventory (BPI) pain scale and 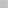 indicates the Least pain in the previous 24 h

**Figure 4:**
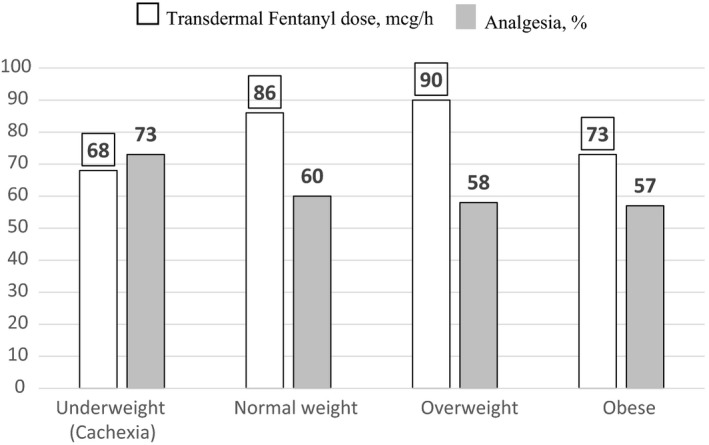
Transdermal Fentanyl dose, mcg/ and % Analgesia as a function of the body mass index (BMI), kg/m^2^ classification used by Center for Disease Control (CDC).^18^ Four BMI categories include underweight or BMI of <18.5, Normal weight or BMI of 18.5 to <25, Overweight or BMI of 25.0 to <30, and obese or BMI ≥30.^19^ □ indicates Transdermal Fentanyl dose, mcg/h and 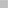 indicates percent Analgesia, % in the previous 24 h

When using BMI classification A,^17^ there were 30 to 70 subjects per each category. Patients with BMI < 20 reported the most pain relief (67%) and lowest Least pain (2.7) (Figure 1), whereas receiving the lowest average TDF dose (72 mcg/h) (Figure 2). This TDF dose approximates the use of 75 mcg TDF patch. When using CDC BMI classification B,^19^ there were 17‐103 per each BMI category. Patients with cachexia reported most pain relief (73%) and lowest Least pain (2.7), whereas receiving lowest TDF dose (68 mcg/h) (Figures 3 and 4). This TDF dose approximates the use of a 62.5 mcg TDF patch. Differences in TDF dose and pain levels were not statistically significant by BMI category.

When using BMI as a continuous variable, correlations with worst pain or least pain were not significant (Tables 2 and 3; *P *≥ .25). Also differences in TDF dose and pain levels were not statistically significant by BMI category. In a regression analysis to predict percent pain relief (Table 4), TDF dose was not significant but BMI category <20 was borderline significantly associated with greater percent pain relief (*P* = .077). When predicting percent pain relief, the BMI category of <18.5 was significantly associated with greater pain relief irrespective of TDF dose (*P* = .038) (Table 5).

**Table 2:** BMI as a continuous variable correlation with Worst or Least pain. Correlation of Continuous BMI with Worst pain in last 24 h

_TYPE_	_NAME_	Worst_pain_24_hrs	BMI
MEAN		6.904	25.538
STD		2.455	5.437
N		240	240
CORR	Worst_pain_24_hrs	1.000	−0.021
CORR	BMI	−0.021	1.000

Note

*P* = .75.

Abbreviation: BMI, body mass index, kg/m^2^.

**Table 3:** BMI as a continuous variable correlation with Worst or Least pain. Correlation of Continuous BMI with Least pain in last 24 h

_TYPE_	_NAME_	Least_pain_24_hrs	BMI
MEAN		3.658	25.538
STD		2.520	5.437
N		240	240
CORR	Least_pain_24_hrs	1.000	0.075
CORR	BMI	0.075	1.000

Note

*P* = .25.

Abbreviation: BMI, body mass index, kg/m^2^.

**Table 4:** Regression analysis to *predict percent pain relief* by BMI classifications A)^17^ and B)^18^. Predicting percent pain relief, using five BMI categories

Obs	Dependent	Parameter	Estimate	StdErr	*t* Value	Prob *t*
1	Percent_relief	Intercept	0.5685303973	0.03595218	15.81	<0.0001
2	Percent_relief	BMICat 20‐21.9	0.0321611071	0.05156682	0.62	0.5335
3	Percent_relief	BMICat 22‐24.9	0.0330409002	0.04848078	0.68	0.4962
4	Percent_relief	BMICat 25‐27.9	0.0356320042	0.04897770	0.73	0.4677
5	**Percent_relief**	**BMICat <20**	**0.1018173991**	**0.05729340**	**1.78**	**0.0769**
6	Percent_relief	BMICat ≥28	0.0000000000	—	—	—
7	Percent_relief	cum_dose	−0.0000648697	0.00024413	−0.27	0.7907

Abbreviation: BMI, body mass index.

Bold values indicate borderline significant.

**Table 5:** Regression analysis to *predict percent pain relief* by BMI classifications A)^17^ and B).^18^ Predicting percent analgesia (pain relief), using four CDC BMI categories

Obs	Dependent	Parameter	Estimate	StdErr	*t* Value	Prob *t*
1	Percent_relief	Intercept	0.5720851071	0.04203762	13.61	<0.0001
2	Percent_relief	BMICat_CDC 18.5‐24.9	0.0269145752	0.04623859	0.58	0.5611
3	Percent_relief	BMICat_CDC 25‐29.9	0.0153329735	0.04916580	0.31	0.7554
4	Percent_relief	BMICat_CDC ≤18.5	0.1561246736	0.07495384	2.08	0.0384
5	Percent_relief	BMICat_CDC ≥30	0.0000000000	—	—	—
6	Percent_relief	cum_dose	−0.0000452082	0.00024235	−0.19	0.8522

Abbreviation: BMI, body mass index.

In regression analysis to predict TDF dose, no BMI categories were statistically significant (*P* > .101). When predicting Least pain in last 24 hours (Table 6) or Worst pain in last 24 hours (Table 7), neither TDF dose nor either categorization of BMI was statistically significant. However, as expected, least and worst pain were strongly inversely associated with percent pain relief.

**Table 6:** Predicting Least pain in last 24 h using (a) five BMI categories and (b) four BMI categories

Dependent	Parameter	Estimate	Biased	StdErr	*t* Value	Prob *t*
(a) Predicting Least pain in last 24 h using five BMI categories						
Least_pain_24_hrs	Intercept	5.918793682	1	0.44812467	13.21	<0.0001
Least_pain_24_hrs	Percent_relief	−4.317997621	0	0.56947592	−7.58	<0.0001
Least_pain_24_hrs	BMICat 20‐21.9	0.332637049	1	0.44476646	0.75	0.4553
Least_pain_24_hrs	BMICat 22‐24.9	0.029915286	1	0.41821799	0.07	0.9430
Least_pain_24_hrs	BMICat 25‐27.9	0.507357652	1	0.42256433	1.20	0.2311
Least_pain_24_hrs	BMICat <20	−0.678202448	1	0.49713223	−1.36	0.1738
Least_pain_24_hrs	BMICat ≥28	0.000000000	1	—	—	—
Least_pain_24_hrs	cum_dose	0.002482152	0	0.00210419	1.18	0.2394

(b) Predicting Least pain in last 24 h using four BMI categories						
Least_pain_24_hrs	Intercept	5.778776563	1	0.49065796	11.78	<0.0001
Least_pain_24_hrs	Percent_relief	−4.310178224	0	0.57281076	−7.52	<0.0001
Least_pain_24_hrs	BMICat_CDC 18.5‐24.9	0.169011616	1	0.40197527	0.42	0.6745
Least_pain_24_hrs	BMICat_CDC 25‐29.9	0.541566336	1	0.42719874	1.27	0.2062
Least_pain_24_hrs	BMICat_CDC ≤18.5	−0.464026636	1	0.65724459	−0.71	0.4809
Least_pain_24_hrs	BMICat_CDC ≥30	0.000000000	1	—	—	—
Least_pain_24_hrs	cum_dose	0.002607455	0	0.00210544	1.24	0.2168

Abbreviation: BMI, body mass index.

**Table 7:** Predicting Worst pain in last 24 h using (a) five BMI categories and (b) four BMI categories

Dependent	Parameter	Estimate	Biased	StdErr	*t* Value	Prob *t*
(a) Predicting Worst pain in last 24 h using five BMI categories						
Worst_pain_24_hrs	Intercept	8.289451717	1	0.47567125	17.43	<0.0001
Worst_pain_24_hrs	Percent_relief	−3.014168179	0	0.60448205	−4.99	<0.0001
Worst_pain_24_hrs	BMICat 20‐21.9	0.308619756	1	0.47210660	0.65	0.5140
Worst_pain_24_hrs	BMICat 22‐24.9	0.271895591	1	0.44392618	0.61	0.5408
Worst_pain_24_hrs	BMICat 25‐27.9	0.152554011	1	0.44853969	0.34	0.7341
Worst_pain_24_hrs	BMICat <20	0.550722322	1	0.52769134	1.04	0.2978
Worst_pain_24_hrs	BMICat ≥28	0.000000000	1	—	—	—
Worst_pain_24_hrs	cum_dose	0.002226626	0	0.00223354	1.00	0.3199

(b) Predicting Worst pain in last 24 h using four BMI categories						
Worst_pain_24_hrs	Intercept	8.005737103	1	0.51659558	15.50	<0.0001
Worst_pain_24_hrs	Percent_relief	−2.943482382	0	0.60309122	−4.88	<0.0001
Worst_pain_24_hrs	BMICat_CDC 18.5‐24.9	0.678625407	1	0.42322487	1.60	0.1102
Worst_pain_24_hrs	BMICat_CDC 25‐29.9	0.538197703	1	0.44978173	1.20	0.2327
Worst_pain_24_hrs	BMICat_CDC ≤18.5	0.242268912	1	0.69198847	0.35	0.7266
Worst_pain_24_hrs	BMICat_CDC ≥30	0.000000000	1	—	—	—
Worst_pain_24_hrs	cum_dose	0.001915757	0	0.00221674	0.86	0.3884

Abbreviation: BMI, body mass index.

Of the covariates of age, cancer diagnosis, and pain etiology (cancer pain vs. cancer treatment‐related pain in a patient with cancer in remission), the only significant covariate associated with higher fentanyl dose was age under 25 (*P* ≤ .004). The only significant covariate associated with greater least pain was cancer of oral cavity and pharynx (*P* ≤ .049) controlling for either four or five BMI categories and other covariates.

## 4 DISCUSSION

Our study is the first prospective clinical effectiveness study to evaluate potential associations between TDF, BMI, and clinical pain outcomes in cancer pain patients. While generally consistent with the American Cancer Society (ACS) and previously published results of all patients in our Pain Registry,^16^ not surprisingly, our patient cohort treated with TDF had a much higher rate of patients with oral cavity, pharyngeal, and laryngeal cancers (8%) compared to the rate of <4% reported by the ACS for the general population.

Low BMI (<18.5) was significantly associated with greater pain relief irrespective of TDF dose and borderline significantly associated with greater percent pain relief after controlling for age, cancer diagnoses, and pain etiology (*P* = .073), suggesting that low BMI may independently predict better pain relief in cancer patients. As there were no significant associations between BMI and TDF dose, we find no basis for BMI‐dependent dose modification or avoiding TDF in cachectic and low BMI patients.

## CONFLICT OF INTEREST

The authors have no conflicts of interest to declare.
